# Timing of Introduction of Complementary Foods — United States, 2016–2018

**DOI:** 10.15585/mmwr.mm6953a1

**Published:** 2023-07-28

**Authors:** Katelyn V. Chiang, Heather C. Hamner, Ruowei Li, Cria G. Perrine

**Affiliations:** ^1^Division of Nutrition, Physical Activity, and Obesity, National Center for Chronic Disease Prevention and Health Promotion, CDC; ^2^Oak Ridge Institute for Science and Education, Oak Ridge, Tennessee.

The American Academy of Pediatrics (AAP) recommends introducing complementary foods (i.e., any solid or liquid other than breast milk or infant formula) to infants at approximately age 6 months (*1*). Although a consensus on ideal timing is lacking, most experts agree that introduction of complementary foods before age 4 months is too early because of infant gastrointestinal and motor immaturity (*1*,*2*). In addition, early introduction prevents exclusively breastfed infants from reaching the recommended 6 months of exclusive breastfeeding (*1*) and might be associated with increased risk for overweight and obesity (*3*). Nationally representative data on complementary feeding are limited; state-level estimates have been previously unavailable. CDC analyzed 2016–2018 data from the National Survey of Children’s Health (NSCH) (N = 23,743) to describe timing of complementary feeding introduction and prevalence of early introduction of complementary foods before age 4 months (early introduction) among children aged 1–5 years. Prevalence of early introduction was 15.6% nationally and varied geographically and across sociodemographic and infant feeding characteristics. These estimates suggest that approximately one in six infants are introduced to complementary foods before they are developmentally ready. Efforts by health care providers and others who might influence infant feeding practices could help decrease the number of infants who are introduced to complementary foods too early.

NSCH is funded and directed by the Maternal and Child Health Bureau of the Health Resources and Services Administration. It is an annual web- and paper-based survey that collects information from parents and caregivers on their children’s physical and emotional health, including infant nutrition, and is representative of noninstitutionalized U.S. children aged 0–17 years. During 2016–2018, the overall weighted response rate ranged from 37.4% to 43.1%. Missing data for race/ethnicity (1.3%) and household income relative to the federal poverty level (FPL) (16.3%) were imputed using hot-deck and sequential regression imputation methods, respectively (*4*).

Timing of introduction of complementary foods was assessed by asking respondents with children aged 0–5 years “How old was this child when he or she was first fed anything other than breast milk or formula” (*4*). To ensure that children had sufficient time to have been introduced to complementary foods, analysis was restricted to children aged 1–5 years. Participants with reported introduction to complementary foods at age >12 months (887) and those with other implausible feeding patterns (recalled breastfeeding duration, infant formula introduction, and complementary feeding introduction indicated ≥2 months with no source of nutrition: 281) were excluded from analyses. The percentage of children who were introduced to complementary foods before age 4 months (early introduction) was calculated overall, at the state and regional levels, and by sociodemographic and infant feeding characteristics using SAS-callable SUDAAN (version 11.0; RTI International). Two-sample t-tests were used to identify statistically significant (p<0.05) differences across subgroups.

Among 23,743 children aged 1–5 years, the mean age at introduction of complementary foods was 5.7 months, with 15.6% of children introduced at <4 months, 63.3% at 4–6 months, and 21.1% at 7–12 months (Figure 1). Prevalence of early introduction varied across sociodemographic groups. Prevalence of early introduction was significantly higher among non-Hispanic Black (Black) children (25.2%), compared with all other racial/ethnic groups, including non-Hispanic other/multiracial children (16.7%), Hispanic children (15.0%), non-Hispanic Asian children (14.4%), and non-Hispanic white children (13.8%). Prevalence of early introduction was significantly lower among children living in households at ≥400% of the FPL (12.1%) and whose mothers had a bachelor’s degree or higher (10.9%), compared with all other household FPL and maternal education groups. The prevalence for early introduction for children living in households at <100% FLP was significantly higher than for those with 200% FLP or higher. Early introduction also differed significantly by infant milk feeding status at age 4 months: prevalence of early introduction was 5.6% among children receiving only breast milk for milk feeds, 14.8% among those receiving breast milk and infant formula, and 23.7% among those receiving only infant formula for milk feeds (Table). At the state level, prevalence of early introduction ranged from 7.6% in New Mexico to 34.4% in Mississippi. In 23 states, the prevalence of children introduced to complementary foods before age 4 months was higher than the national prevalence (15.6%), including four states in which prevalence of early introduction was at least 20% (Supplementary Table, https://stacks.cdc.gov/view/cdc/131235) (Figure 2).

**Figure 1:**
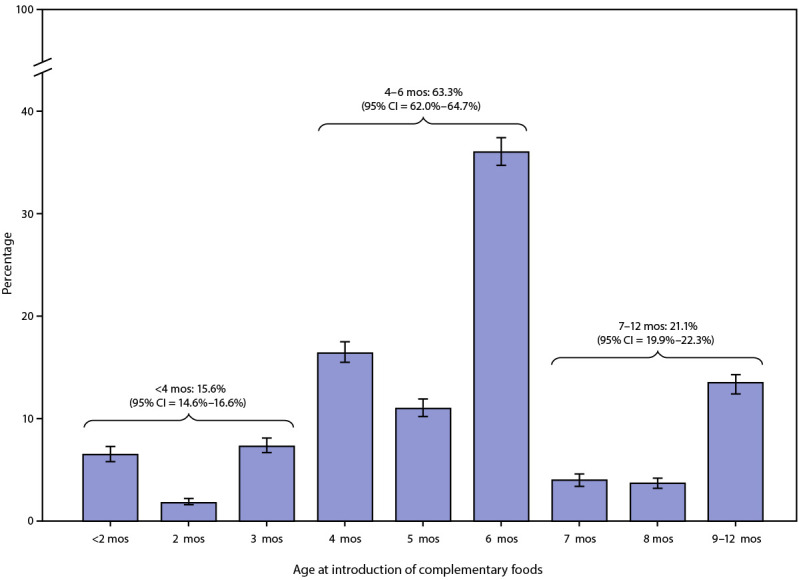
Age at introduction of complementary foods among children aged 1–5 years* — National Survey of Children's Health, United States, 2016–2018 **Abbreviation:** CI = confidence interval. * 95% confidence intervals are indicated by error bars.

**TABLE:** Percentage of infants introduced to complementary foods before age 4 months, by sociodemographic characteristics, infant milk feeding status at age 4 months, and region among children aged 1–5 years — National Survey of Children's Health, United States, 2016–2018

Characteristic	Total no.*	% Introduced early^†^	95% CI^†^
**Total**	**23,743**	**15.6%**	**(14.6–16.6)**
**Race/Ethnicity^§^**			
Hispanic	2,605	15.0	(12.4–18.0)
White, non-Hispanic	16,721	13.8	(12.9–14.8)
Black, non-Hispanic	1,206	25.2	(21.2–29.6)
Asian, non-Hispanic	1,115	14.4	(10.7–19.2)
Other/Multiracial, non-Hispanic	2,096	16.7	(13.8–20.1)
Maternal age group (yrs)^¶,^**			
18–29	4,604	15.8	(13.8–17.9)
30–39	14,099	12.4	(11.2–13.6)
≥40	3,390	17.4	(14.8–20.3)
**Maternal highest education level^¶,^** ^††^			
High school diploma or less	2,526	18.4	(15.6–21.5)
Some college	5,920	15.8	(14.0–17.8)
Bachelor's degree or more	13,569	10.9	(9.9–12.0)
**Household income^§§^**			
<100% FPL	2,405	19.8	(16.8–23.2)
100%–199% FPL	3,719	18.2	(15.4–21.4)
200%–399% FPL	7,608	14.8	(13.0–16.7)
≥400% FPL	10,012	12.1	(10.8–13.4)
**Infant milk feeding status at age 4 mos^¶¶^**			
Breast milk feeding only	9,109	5.6	(4.7–6.8)
Infant formula feeding only	8,667	23.7	(21.9–25.6)
Mixed breast milk and infant formula feeding	5,750	14.8	(12.8–17.1)
Region***^,^ ^†††^			
Northeast	4,066	16.7	(14.3–19.4)
Midwest	6,020	15.2	(13.6–16.9)
South	7,613	17.8	(16.2–19.7)
West	6,044	11.6	(9.8–13.8)

**Abbreviations:** CI = confidence interval; FPL = federal poverty level.

* Denominators might not sum to total because of missing maternal sociodemographic or infant milk feeding status data.

^†^ Percentages are weighted to account for complex survey design.

^§^ The percentage of infants introduced to complementary foods before age 4 months among non-Hispanic Black children is significantly different from that of non-Hispanic White children, non-Hispanic Asian children, Hispanic children, and non-Hispanic other/multiracial children.

^¶^ Maternal sociodemographic data might be missing because no mother was reported in the child’s household or because information was not reported by respondent.

** The percentage of infants introduced to complementary foods before age 4 months among children of mothers aged 30–39 years is significantly different from that of children of mothers aged 18–29 and ≥40 years.

^††^ The percentage of infants introduced to complementary foods before 4 months among children of mothers with bachelor’s degrees or higher is significantly different from that of children of mothers of all other highest education levels.

^§§^ The percentage of infants introduced to complementary foods before 4 months among children living at ≥400% FPL is significantly different from that of children living at all other household income levels. The percentage of infants introduced early among children living at <100% FPL is significantly different from that of children living at 200%–399% FPL and ≥400% FPL.

^¶¶^ The percentage of infants introduced to complementary foods before age 4 months among children receiving only breast milk for milk feeds at age 4 months is significantly different from that of children receiving all other types of nutrition for milk feeds at age 4 months. The percentage introduced early among children receiving only infant formula for milk feeds at age 4 months is significantly different from that of children receiving all other types of nutrition for milk feeds at age 4 months. The percentage introduced early among children receiving both breast milk and infant formula for milk feeds at age 4 months is significantly different from that of children receiving all other types of nutrition for milk feeds at age 4 months.

*** U.S. Census Bureau classifications for regions.

^†††^ The percentage of children introduced to complementary foods before age 4 months among children living in the West is significantly different from that of children living in all other regions. The percentage of children introduced early among children living in the Midwest is significantly different from that of children living in the South and the West.

**Figure 2:**
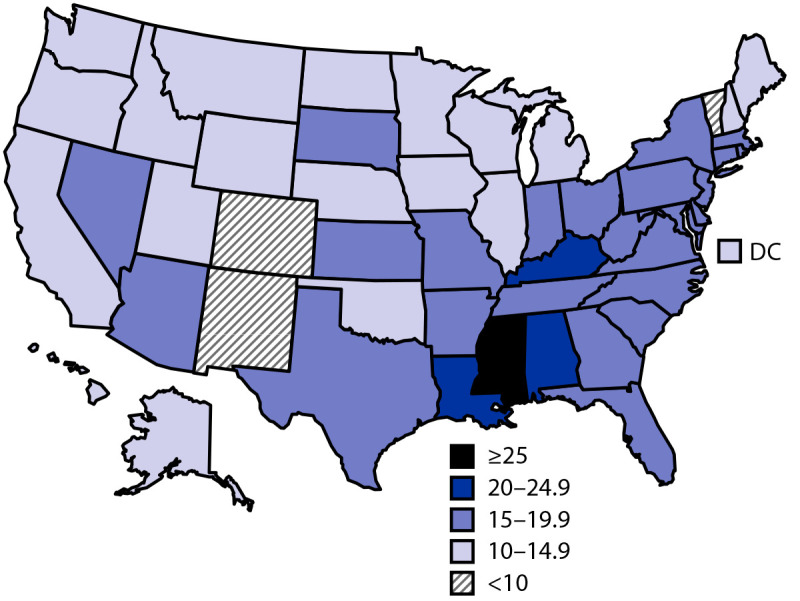
Percentage of children introduced to complementary foods before age 4 months among children aged 1–5 years — National Survey of Children's Health, United States, 2016–2018 **Abbreviation**: DC = District of Columbia.

## Discussion

Approximately one in six (15.6%) U.S. infants is introduced to complementary foods before age 4 months, with a higher prevalence of early introduction among Black infants and infants of mothers and households at lower socioeconomic status. Reasons for early introduction to complementary foods are not fully understood; however, many early introducing mothers have reported believing that their infant was old enough to begin consuming solids (*5*). This suggests a perception of infant readiness for complementary feeding before the infant is actually ready and a potential lack of awareness of feeding recommendations, health effects associated with early introduction, and signs of developmental readiness. In general, infants show outward signs of readiness for complementary feeding when they can sit up on their own with good head control, show interest in mealtimes, are hungry in between feedings, and no longer have “tongue-thrust” or extrusion reflex, usually at approximately age 4–6 months (*2*).

Not only do younger infants lack the physiologic development to safely consume complementary foods, infants who are introduced to complementary foods too early have increased risk for multiple associated health conditions (*1*). Early introduction to complementary foods prevents infants from meeting the recommended 6 months of exclusive breastfeeding, decreasing the benefits both mothers and infants derive from exclusive breastfeeding. Compared with exclusive breastfeeding for 6 months, exclusive breastfeeding for 3–4 months followed by mixed breastfeeding and complementary feeding is associated with increased risk for gastrointestinal infection and slower maternal weight loss after birth (*6*). Further, limited evidence also suggests introduction to complementary foods before age 4 months might increase later overweight and obesity risk (*3*).

Health care providers can help increase awareness of recommended timing of introduction of complementary foods by employing consistent messaging in accordance with AAP recommendations and stressing the importance of developmental readiness when discussing complementary feeding with families (*1*). Resources are available to help health care providers engage with and educate families to better navigate the transition from milk feeds to family foods (*7*). Further, given the higher prevalence of early introduction of complementary foods among infants receiving formula, targeted education to parents and caregivers of those receiving infant formula might be particularly helpful. Similar efforts by others who could influence infant feeding practices such as peer educators, early care and education staff members, and Special Supplemental Nutrition Program for Women, Infants, and Children (WIC) staff members might also help reduce early introduction.

Another nationally representative study of U.S. children, the 2009–2014 National Health and Nutrition Examination Surveys (NHANES), found a similar prevalence of early introduction (16.3%); in addition, similar patterns in early introduction by sociodemographic and infant feeding status characteristics were seen across both studies (*8*). The questions used to identify timing of complementary feeding introduction were the same for both studies. These findings, that most infants are not introduced to complementary foods early, might indicate that parents, caregivers, and health care providers have been receptive to early food introduction recommendations. Continued education and clear communication on appropriate timing is important.

The findings in this report are subject to at least five limitations. First, an unexpected clustering of reported month of introduction at exactly 12 months was observed. Approximately 7.4% of the sample reported 12 months versus 1.2% at 11 months and 0.5% at 13 months. It was hypothesized that the clustering might be rounding from nearby categories but that respondents likely introduced complementary foods late in the first year. A sensitivity analysis excluded those who reported ≥12 months from the denominator, because of the potential implausibility of these responses, and found the prevalence of early introduction increased by 1.2 percentage points. Second, data might be affected by information bias. Though maternal recall of breastfeeding has been shown to have high validity and reliability, recall of solid and other liquid feeding might not be as reliable (*9*). However, participants with implausible feeding patterns were removed from the sample to account for potential misreporting of infant feeding information. Third, although multiply imputed, household FPL data might be misclassified. Fourth, data do not allow for analysis of types, amounts, or frequency of complementary foods offered; these are important markers of early child nutrition. Finally, small sample sizes limited the ability to conduct further sociodemographic analyses at the state level.

Introduction of complementary foods at the recommended time could help improve infant health and might play a role in prevention of overweight and obesity; however, nearly one in six infants are introduced to complementary foods too early. Early introduction also varies geographically and across sociodemographic and infant feeding characteristics, placing some infants, such as Black infants and infants of mothers and households of lower socioeconomic status, at increased risk for potential poor health outcomes related to early introduction of complementary foods. Increased education on complementary feeding recommendations, including the possible effects of early introduction and signs of developmental readiness, might help decrease the number of infants who are introduced to complementary foods too early.

**Summary**

**What is already known about this topic?**

The American Academy of Pediatrics recommends introducing complementary foods at approximately age 6 months. Introduction before age 4 months is too early because infants are not developmentally ready for complementary foods. Early introduction prevents infants from reaching the recommended 6 months of exclusive breastfeeding.

**What is added by this report?**

Nearly one in six infants is introduced to complementary foods before age 4 months; prevalence of early introduction varies geographically and across sociodemographic and infant feeding characteristics.

**What are the implications for public health practice?**

Increasing awareness of and adherence to feeding recommendations could help reduce early introduction. Health care providers and others who might influence infant feeding practices should educate families on recommended timing of introduction of complementary foods.
